# Connecting Skin and Gut in Dermatitis Herpetiformis: Insights from Gluten Challenge Transcriptomics

**DOI:** 10.2340/actadv.v106.adv-2026-0792

**Published:** 2026-09-09

**Authors:** Helka Kaunisto, Esko Kemppainen, Teea Salmi, Katarzyna Leskinen, Heli Pessa, Rebecka Ventin-Holmberg, Päivi Saavalainen, Kaisa Hervonen, Tuire Ilus, Katri Kaukinen, Katri Lindfors

**Affiliations:** 1 Celiac Disease Research Center, Faculty of Medicine and Health Technology, Tampere University, Tampere, Finland; 2 Department of Dermatology, Tampere University Hospital, Wellbeing services county of Pirkanmaa, Tampere, Finland; 3 Folkhälsan Research Center, Biomedicum, Helsinki, Finland; 4 Immunomics Research Group, Faculty of Medicine, University of Helsinki, Helsinki, Finland; 5 Department of internal Medicine, Tampere University Hospital, Wellbeing services county of Pirkanmaa, Tampere, Finland

**Keywords:** dermatitis herpetiformis, transcriptome, autoimmune disease

## Abstract

Dermatitis herpetiformis is a dietary gluten-induced autoimmune blistering skin disease. It is hypothesized to be initiated by a small intestinal mucosal immune response. Gluten-free diet is the primary treatment for dermatitis herpetiformis and effectively resolves skin lesions. Previous studies show that after gluten reintroduction, peripheral cell responses are detectable already in 6 days and most patients relapse clinically within months. The relapse presentation may differ between patients, with some experiencing both rash and small intestinal mucosal damage and some only one of these. Here, RNA transcriptomics were utilized to characterize temporal systemic and small intestinal mucosal responses to gluten in dermatitis herpetiformis and to identify transcriptomic profiles specific for rash or villous atrophy as relapse presentations. The cohort consisted of 19 dermatitis herpetiformis patients on gluten-free diet who underwent a gluten challenge. Gluten challenge elicited pronounced gene expression changes related to inflammation in the small intestine. In patients relapsing with rash, *DOCK8* expression was significantly upregulated, while *IL10RB* was downregulated at end of follow-up. These patients also had significantly increased frequency of intraepithelial lymphocytes. Our findings show that prolonged gluten exposure alters duodenal transcriptomic profiles considerably. Moreover, patterns related to rash could be detected and present interesting candidates for future studies.

**SIGNIFICANCE**

In this study, we examined how gluten affects gene activity in blood cells and small intestine in the chronic, autoimmune blistering skin disease dermatitis herpetiformis. We found that genes related to inflammation and immune activity were switched on, while genes involved in nutrient absorption were switched off. Although the overall intestinal response was similar across patients, important differences emerged depending on whether the patients presented with rash or intestinal damage after gluten consumption. These findings support the idea that immune reactions in the small intestine play a central role in dermatitis herpetiformis and help explain why dermatitis herpetiformis can present differently between individuals.

Dermatitis herpetiformis (DH) is a dietary gluten-driven blistering skin disease regarded as an extraintestinal manifestation of celiac disease (CeD). A hallmark of DH is the deposition of IgA in perilesional skin, which along with clinical symptoms, such as intensive itch and blisters, forms the basis of diagnosis (1). The IgA depositions co-localize with epidermal transglutaminase (transglutaminase 3, TG3) which is considered the main autoantigen in DH (2). Patients also have serum IgA antibodies targeting TG3 (3) and TG3-specific antibody-producing plasma cells have been identified in the small intestinal mucosa of patients (4, 5), linking the gut immune response to the cutaneous manifestations. Approximately 75% of DH patients display villous atrophy. The remainder, along with those with atrophy, have increased densities of specialized T cells (intraepithelial lymphocytes (IELs)), paralleling potential CeD with normal small intestinal morphology but positive CeD autoantibodies (6). Accordingly, the small intestine appears as an integral site for DH and in fact has been proposed as the initiation site for the disease (7).

The treatment for DH is a strict life-long gluten free diet, which heals the skin symptoms and small intestinal damage. However, upon reintroduction of gluten, most patients relapse within months (8–12). Curiously, the relapse may present differently in individual patients, with some experiencing both rash and villous atrophy and some only with either. Noteworthily, even a short 3-day gluten challenge induces gluten-specific T cell response in the periphery in a subset of patients, similarly to CeD (13, 14). While the effects of long and short gluten challenge on small intestinal mucosal and peripheral immune cell RNA expression have been characterized in CeD (15, 16), corresponding information in DH is lacking. In addition, it is not understood why the relapse presentation to extended gluten challenge in DH patients varies.

The aim of this study was to investigate the temporal transcriptomic changes induced by a short-term gluten challenge in the peripheral blood, followed by effects of long-term gluten challenge in the small intestine. A further aim was to identify intestinal mucosal transcriptomic changes associated with the development of rash or villous atrophy as different relapse presentations.

## MATERIALS AND METHODS

### Study patients

The study cohort, sample collection, and methods for dermal IgA and IELs have been described previously (12). The study protocol was approved by the Ethics Committee of Pirkanmaa hospital district (R16039), and all study participants gave written informed consent. Peripheral blood mononuclear cells (PBMCs) and small intestinal biopsies were collected from DH patients in remission, on day 0. Thereafter, the patients consumed 200 g wheat-containing bread for 3 days, followed by exclusion of gluten for 3 days. On day 6, PBMCs were sampled (17). Patients resumed gluten consumption (≥10 g/day) until relapse or a maximum of 12 months when the follow-up ended. Table SI summarizes key characteristics of patients included in differential gene expression analysis. The median sampling time for end of follow-up biopsies was 9 months (range 1–12 months).

### Bulk RNA sequencing library construction and analysis

Total RNA was extracted from cryopreserved PBMCs using the RNeasy Mini kit (Qiagen). The RNAlater preserved intestinal biopsies were homogenized using bead-beating a FastPrep Tissue homogenizer followed by RNA purification with RNeasy Mini kit (Qiagen). The libraries were prepared based on a modified Drop-seq protocol (18), as described (19, 20). Genes with false discovery rate (FDR)<0.05 and log2 fold change ≥1 or ≤ –1 were considered significantly differentially expressed. The differential gene expression analysis was conducted temporally, comparing day 0 and follow-up samples unless otherwise described. A detailed description of the library construction, data processing and tools used for downstream analyses is presented in Appendix S1, and sequences for primers and indexes used in sequencing in Table SII.

### Statistical analyses

Statistical analyses were carried out in RStudio. Differences between means of groups were tested with either unpaired t-test or Wilcoxon rank-sum test.

## RESULTS

### Short gluten challenge elicits modest transcriptomic changes in PBMCs

**Fig. 1.**
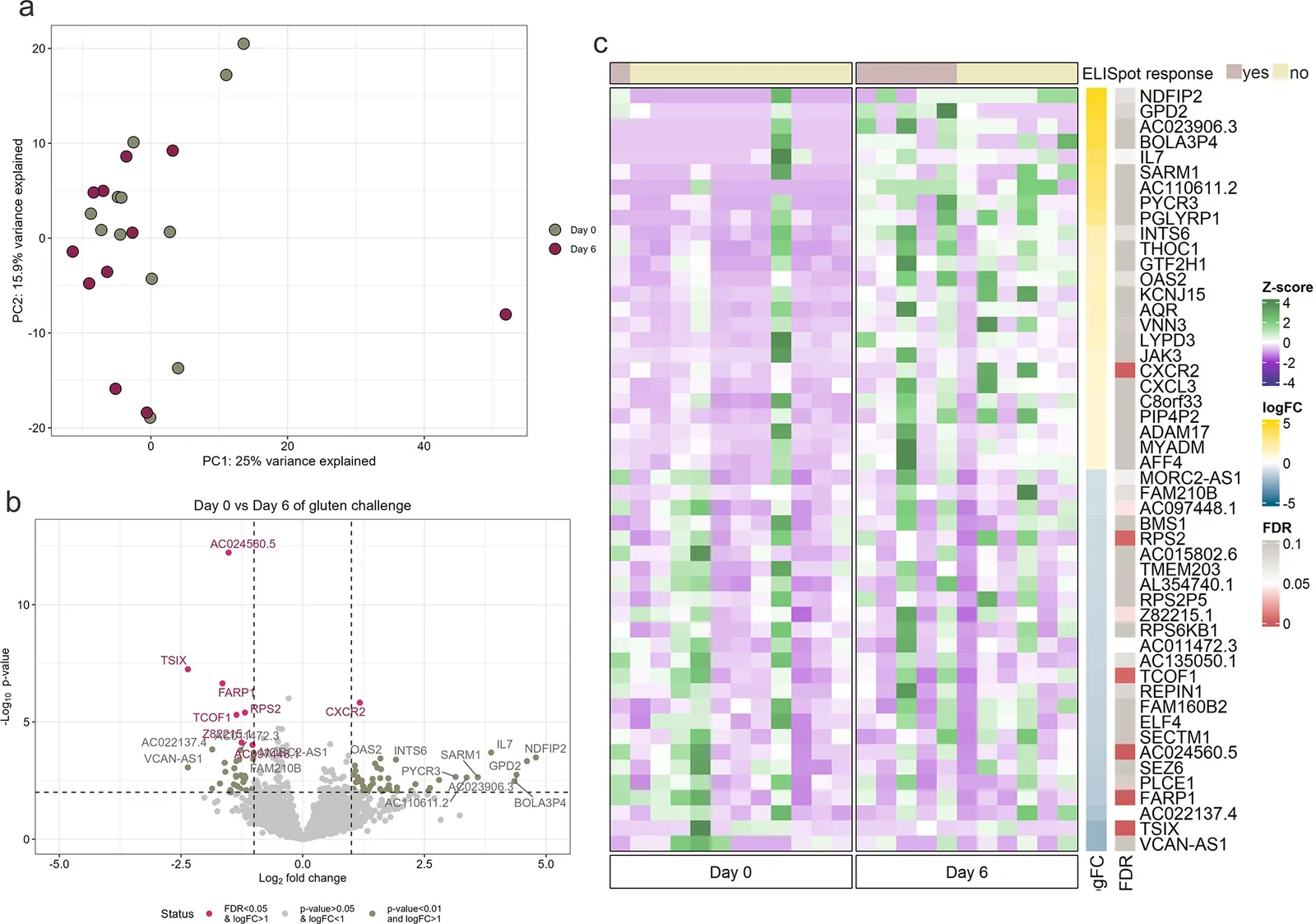
Effects of a short, high dose gluten challenge on dermatitis herpetiformis patient peripheral blood mononuclear cells gene expression. (a) Principal component analysis shows no clear clustering of day 0 and day 6 samples. (b) Volcano plot of up and downregulated genes on day 6 of gluten challenge, genes with FDR<0.05 and log2 fold change ≥1 were considered significantly differentially expressed and are denoted in red. Genes with *p*-value<0.01 and log2 fold change ≥1 denoted in pale green. (c) Heatmap of the top 25 altered genes in both directions (*p*-value <0.01). Genes are ranked in order of log2 fold change, and FDR value is displayed on the right. Positive interferon-γ response on day 6 to gliadin in ELISpot is indicated on the bar above the heatmaps. PC, principal component; FDR, false discovery rate.

Principal component analysis (PCA) of PBMC transcriptome of all the available samples on day 0 before and day 6 after the 3-day gluten challenge showed no clear clustering and only modest changes in gene expression (**Fig. 1**). The only significantly upregulated gene on day 6 was the interleukin-8 receptor gene *CXCR2* (Fig. 1b, c). In addition, although not resistant to correction for multiple comparisons, several immune-related genes (*IL7, CXCL3, CXCL2, JAK3*) were upregulated (Fig. 1c and Table SIII). Four noncoding RNAs, as well as protein-coding genes *FARP1*, *TCOF1* and *RPS2,* were significantly downregulated on day 6 (Fig. 1b, c). Of the patients with PBMC transcriptome data available, 5 were previously shown to mount a peripheral gluten-specific T cell response (13), but they neither separated from nonreactive patients in PCA (data not shown) nor shared a common differential gene expression profile (Fig. 1c).

### Duodenal transcriptomic responses to gluten challenge and signatures of mucosal damage

**Fig. 2.**
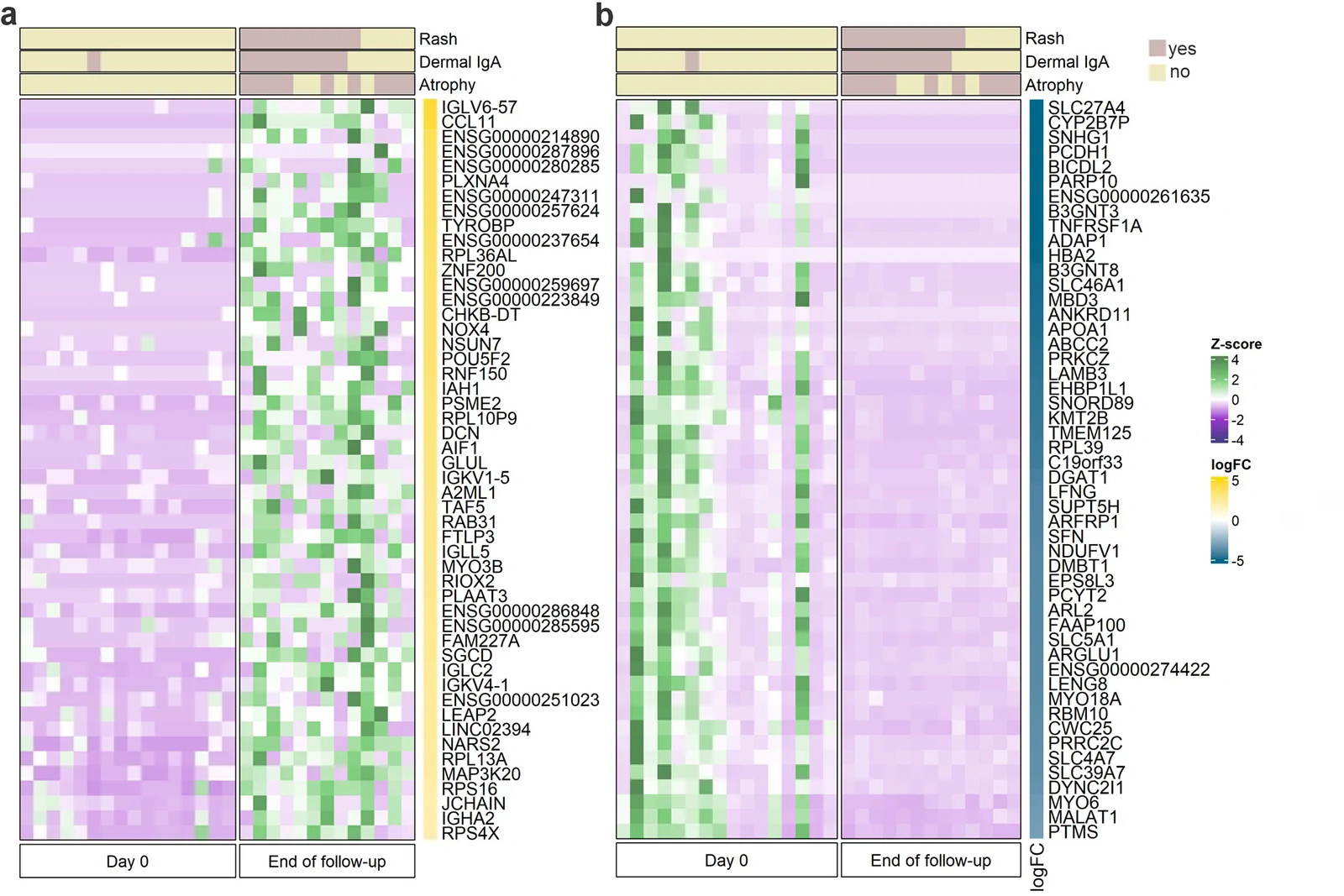
Changes in gene expression profiles in dermatitis herpetiformis patient duodenal biopsies in response to a long gluten challenge. (A) Heatmap of the top 50 upregulated (A) and downregulated (B) genes at FE. The presence of rash, dermal IgA deposition and small intestinal atrophy are indicated on the bars above the heatmaps. logFC, log2 fold change; FE, end of follow-up.

**Fig. 3.**
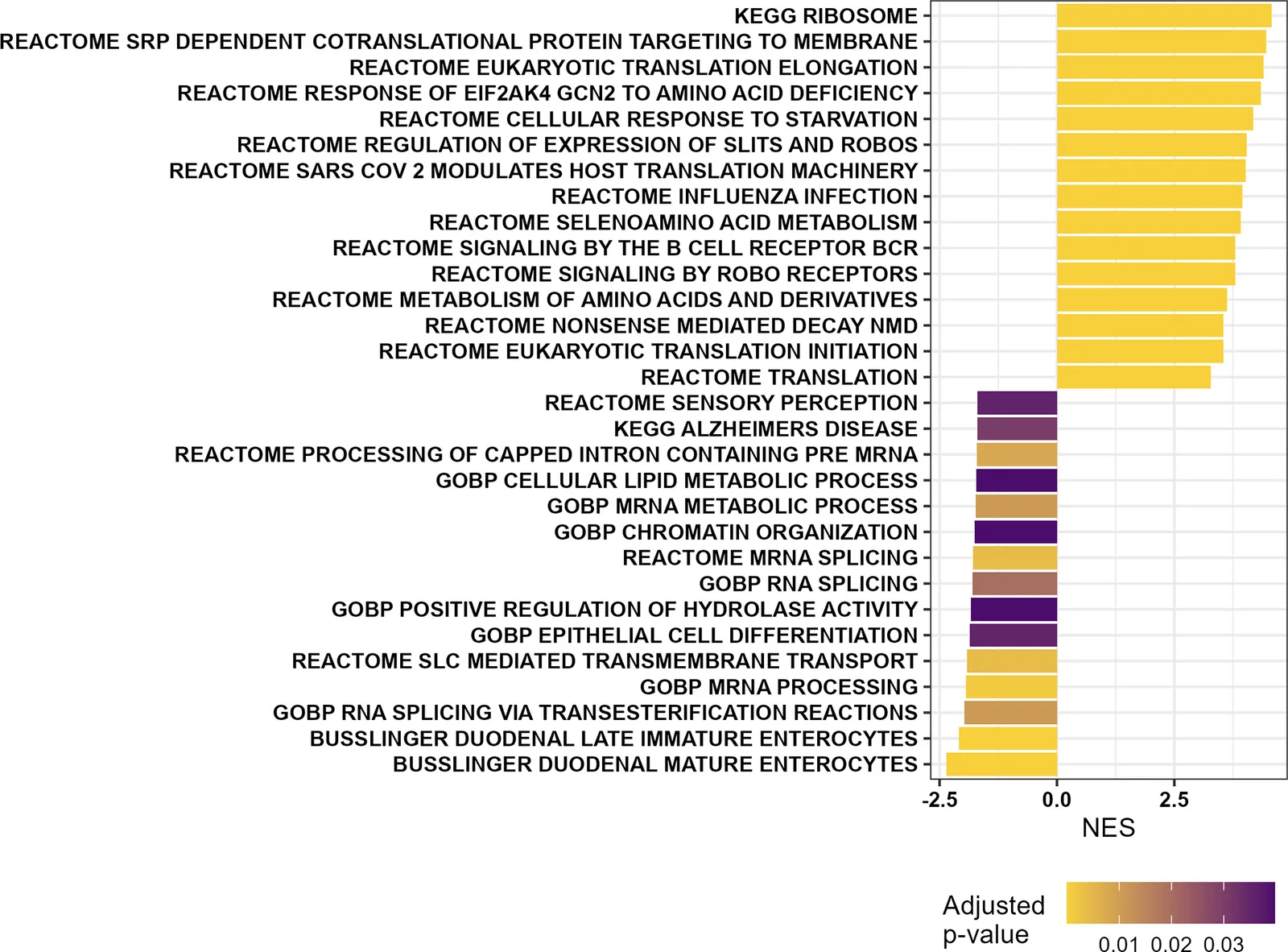
Top 30 significantly enriched gene sets derived from differential gene expression analysis of dermatitis herpetiformis patient duodenal biopsies. Gene set enrichment analysis of the significantly differentially expressed genes, showing the 15 most enriched gene sets at end of follow-up and on day 0 of gluten challenge (NES>0 and NES<0, respectively). NES, normalized enrichment score.

In the small intestinal mucosal transcriptome analysis, there was a clear distinction between day 0 and follow-up samples in PCA (Fig. S1). Altogether 2016 significantly differentially expressed genes (DEGs) were identified between day 0 and follow-up samples (Table SIV). Among the 545 upregulated genes at follow-up were several related to immune activation (*CCL11, STAT1, IFITM3),* immunoglobulins (such as *JCHAIN, IGHA2, IGKV4-1, IGKV3-20* and *IGLV1-40*) and increased protein transcription and DNA translation (ribosomal proteins, *EIF3M, EIF3K*). In contrast, genes related to epithelial cell differentiation and maturity (*APOA1, APOA4, APOB, APOC3, SI, TNFRSF1A, EZR*) and nutrient transport (*SLC27A4, SLC46A1, SLC5A1, SLC7A8*) were downregulated in follow-up samples. The top 50 up- and downregulated genes are listed in **Fig. 2**a and b. Gene set enrichment analysis (GSEA) of DEGs showed enrichment of B cell receptor signalling, stem cells, transit amplifying zone cells and oxidative phosphorylation in follow-up samples, whereas lipid metabolism, epithelial differentiation and solute carrier mediated transport were enriched on day 0 (**Fig. 3** and Table SV). All pathways are listed in Table SV.

**Fig. 4.**
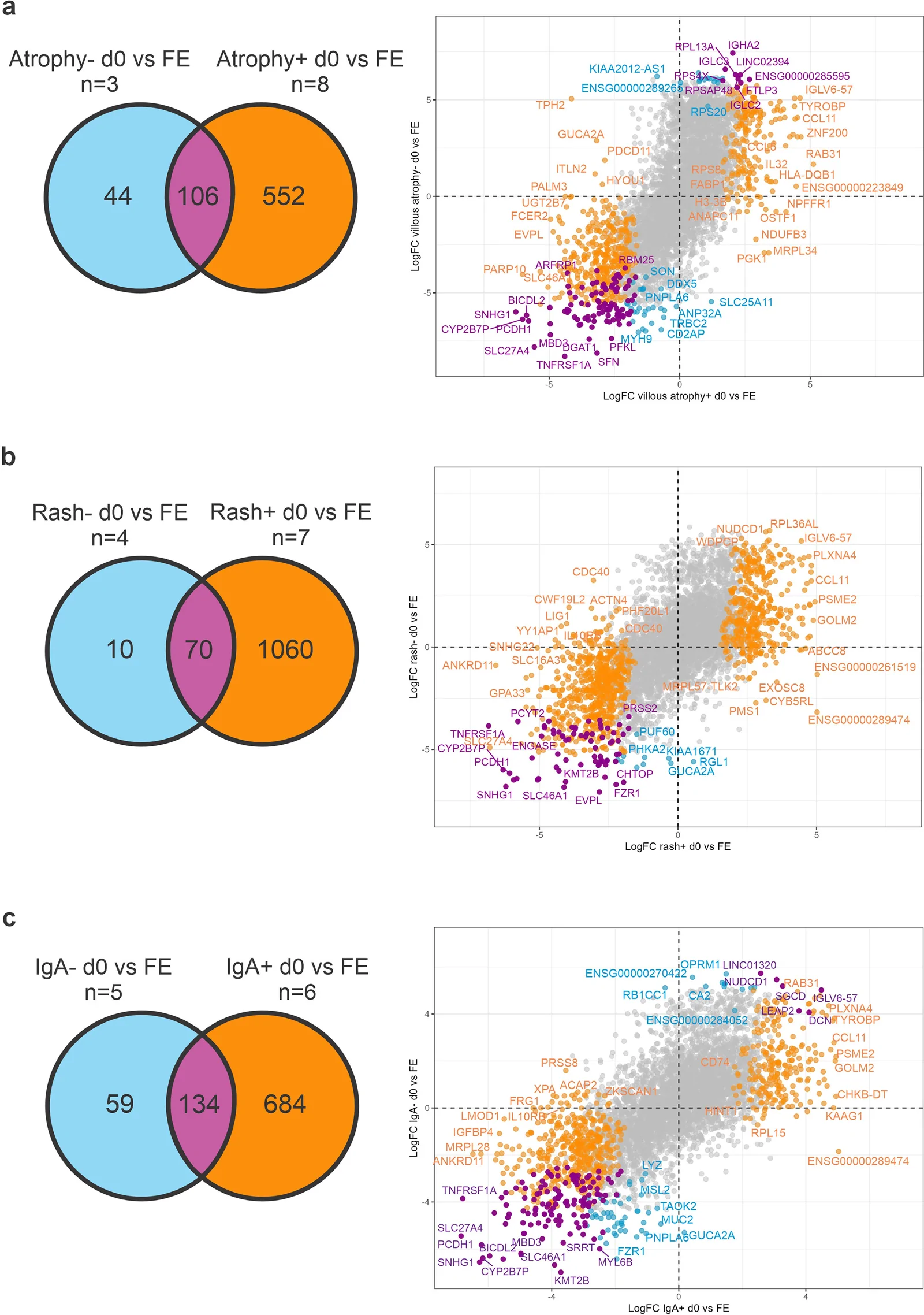
Differential gene expression in paired comparisons of intestinal biopsies between day 0 and end of follow-up (FE) of patients with different relapse responses; presence or absence of small intestinal villous atrophy (A), rash (B), and dermal IgA deposition (C). In both the Venn diagrams and scatter plots, genes that are significantly differentially expressed in both groups are highlighted in purple, genes significant in the positive response group are highlighted in orange, and genes significant in the negative response group are highlighted in blue. In the scatter plots, the x-axes display log2 fold changes of genes between day 0 and FE in patients in positive (atrophy+, rash+, or IgA+) response group, while the y-axes display the negative (atrophy-, rash-, or IgA-) response group. d0, day 0; FE, end of follow-up; logFC, log2 fold change.

At follow-up, 9 patients had developed small-intestinal mucosal damage (villous height-to-crypt depth ratio (Vh:CrD)<2) as relapse presentation, while 4 had normal duodenal morphology (Vh:CrD>2). On day 0, Vh:CrD (median 2.75 vs 2.5) and IEL densities (median CD3^+^ cells 40 and 36, γδ^+^ cells 8.4 and 7.5) were comparable between patients with and without atrophy, and there were no differences in the small intestinal gene expression (data not shown). To examine transcriptomic changes associated with the development of atrophy, patients were stratified according to the presence or absence of atrophy at follow-up. Gene-expression profiles from day 0 to follow-up were analysed separately within each group and thereafter, the profiles were compared to identify patterns linked to the development of atrophy. To minimize the effect of intersubject variability on the results, only data from paired patients (both day 0 and follow-up samples available) were included, resulting in 8 subjects with and 3 without villous atrophy at follow-up (**Fig. 4**a). In patients with villous atrophy, there were 552 unique DEGs, and among patients without atrophy, 44 unique DEGs. Between groups, 106 DEGs were shared (Fig. 4a, Tables SVI and SVII). GSEA revealed negative enrichment of mature enterocyte signatures and positive enrichment of transit-amplifying cell signatures in patients with villous atrophy (Table SVIII), whereas only amide metabolic processes and duodenal mature enterocytes were altered in those without atrophy (Table SIX). Among the 552 DEGs specific to patients with villous atrophy at follow-up, 29 upregulated DEGs were not differentially expressed in the general gluten challenge analysis, including *HLA-DQB1, IL-32* and *CCL5* (Table SVI). Out of the 150 DEGs in patients without villous atrophy, 9 (*NALCN, ENSG00000289369, SERP1, SH3TC2, CMC2, KIAA2012-AS1, DIS3, ENSG00000289265, SLC24A11*) were not significantly differentially expressed in patients with atrophy or in the general gluten challenge (Fig. 4a and Table SVII).

Most DEGs in patients with and without atrophy at follow-up showed comparable log-fold-change magnitudes and directions, despite not always reaching significance in both groups. Consistent with this, the cross-sectional comparison of follow-up samples between patients with and without atrophy did not yield any statistically significant DEGs (data not shown). Therefore, to identify the genes that exhibit genuine differences between patients with or without atrophy, we filtered the DEGs to those with logFC>2 or <-2 in patients with and logFC<1 or >-1 without atrophy. This analysis for diverging expression revealed 73 genes (Table SX). Notably, *HLA-DQB1* and *HLA-DRB5* were significantly upregulated in patients with atrophy (logFC 3.6 and 2.6, respectively) but not in patients without atrophy (logFC 0.7 and 0.8), while conversely *GUCA2A* and *CMC2*, among others, were significantly upregulated only in patients without atrophy (Table SX).

### Gluten challenge induces intestinal changes associated with rash or dermal IgA depositions

For analysis of temporal gene expression patterns related to dermal relapse outcomes, paired samples were used. At follow-up, 7 patients developed rash and 4 did not.

In patients with rash at follow-up, altogether 1,130 significant gluten-responsive DEGs were identified, which, when subjected to GSEA, yielded 42 enriched gene sets (Table SXI and SXIII) that closely mirrored those found in the overall gluten challenge cohort. In patients without rash at follow-up, 80 DEGs were identified (Fig. 4b, Table SXII). When assessing genes with truly diverging expression between patients with or without rash, exploiting the log fold change criteria described above, a total of 270 genes specific for rash-associated response were found. Among these, the expression of immune-related genes *CD84, C8A, HLA-DPA1*, *HLA-DRB1, MS4A2* (FcεR1) and *DOCK8* were significantly upregulated at follow-up in patients with rash, while *IL10RB* and *IL18BP* were downregulated (Table XIV). Only 3 genes (*KIAA1671, GUCA2,* and *RGL1*) were significantly downregulated exclusively in patients without rash (Fig. 4b and Table SXIV).

Except for one patient, all individuals with rash at follow-up also exhibited dermal IgA deposition. Comparison of paired day 0 and follow-up samples from patients with (*n*=6) or without (*n*=5) dermal IgA deposition at follow-up, altogether 818 DEGs were found; 684 unique in patients with IgA deposits, 59 unique in patients without IgA deposits, and 134 shared (Fig. 4c and Table SXV). From DEGs found in dermal IgA positive patients at follow-up, 28 significantly enriched gene sets were identified (Table SXVII, for comparison DEGs in dermal IgA negative patients in Table XVII). Out of the 818 DEGs in the IgA positive patients, only 18 were not among those found in patients with rash at follow-up. Nine of these were unique to IgA deposition positive patients (*TMEM106C, RIMS2, ZKSCAN1, AP1B1, ENSG00000236451, QSOX1, DHX9, COL1A2, ZSWIM8*), and 9 to deposit negative subjects (*MUC2, ENSG00000270442, ELP1, LCTL, PRKRA, ENSG00000261227, RB1CC1, ORC4, CA2*).

Upon conducting the diverging gene expression analysis between dermal IgA-positive and negative patients with the stringent log-fold change criteria, we identified 162 genes that met the thresholds. Most of these were the same genes with identical directional expression patterns as found in analysis between patients with and without rash, such as *IL10RB, CD84, HLA-DPA1*, *DOCK8,* and *GUCA2* (Table SXVIII). In addition, there were 54 DEGs that were not found in the rash at follow-up analysis, including *EPCAM* and *EIF3K*, but none directly related to immune responses (Table SXVIII).

To further elucidate the possible connection between the dermal relapse responses and the small intestine, associations between mucosal morphology, IEL counts (previously shown to increase at follow-up (12)), and rash or dermal IgA deposition were assessed using all available samples (*n*=19). No significant differences were observed in Vh:CrD on day 0 or at follow-up between patients with or without rash, nor between those with or without IgA deposition (**Table I**). IEL counts on day 0 showed no association (data not shown) but at follow-up, patients with rash had significantly higher CD3^+^ and γδ^+^ cell counts (*p*-value 0.0382 and 0.0346, respectively, Table I), than patients without. In patients with dermal IgA at follow-up, γδ^+^ cell counts were significantly higher compared to patients without the deposits (*p*-value 0.0182), while total CD3^+^ T cell counts were not significantly different (Table I).

**Table I.** Lymphocyte count and mucosal morphology at end of follow-up in relation to dermatological and small intestinal characteristics

	Rash		Dermal IgA		Villous atrophy	
	Present, *n*=15 Median (IQR)	Absent, *n*=4 Median (IQR)	Present, *n*=13 Median (IQR)	Absent, *n*=6 Median (IQR)	Present, *n*=15 Median (IQR)	Absent, *n*=4 Median (IQR)
**Vh:CrD**	0.8 (0.6–1.4)	0.9 (0.8–1.5)	0.8 (0.7–1.5)	0.9 (0.8–1)	**0.8** (0.55–1)	**2.55** (2.45–2.72)
IEL counts*						
CD3^+^ cells	**83.5** (60-107)	**44.5** (36-59)	83 (59–103)	65 (40–78)	77 (53.5–105)	60 (47.5–75.5)
γδ ^+^ cells	**17.2** (11-24)	**8.9** (4-13)	**12** (14–26)	**6** (6-14)	14.8 (9.05–19.2)	18.2 (9.85–28.2)

Significant results (*p*<0.05) highlighted in bold.

*n=18 for IEL counts. Unpaired *t*-test for CD3+ IEL cells, Wilcoxon rank sum test for Vh:CrD and γδ + IEL cells.

IEL: intra-epithelial lymphocyte; IQR: interquartile range; Vh:CrD: villous height-to-crypt depth ratio.

## DISCUSSION

In this study, we present transcriptomic analysis of DH patients’ PBMCs and small intestinal biopsies in response to a gluten challenge, and further extend the analysis to subgroup analyses of different relapse responses.

Transcriptomics have previously not been applied in studying the small intestine or PBMCs of DH patients, and only one microarray study of DH skin exists to date, making this study the first of its kind (21). Our results show that DH patient PBMCs exhibited only minimal transcriptomic changes during a short, high-dose gluten challenge, contrasting previous results from CeD patients where a similar short gluten challenge induced a large-scale upregulation of immune genes (15). Although this discrepancy could suggest specific differences in PBMC responses to gluten challenge between DH and CeD patients, this seems unlikely given previously reported comparable interferon-γ ELISpot responses (13), and similar anti-gliadin T-cell phenotypes in both conditions (14). These observations, together with the fact that gluten-specific T cells constitute<1% of circulating cells on day 6 (14), support our findings and suggest that gluten induced transcriptome changes in DH peripheral blood are subtle at the scale of entire PBMC transcriptome and mirror those reported in cross-sectional studies of CeD (22).

In contrast, the small intestinal transcriptomic response of DH patients to gluten challenge closely paralleled that reported in CeD (16), with upregulation of immunoglobulin genes and downregulation of genes involved in nutrient transport and mature epithelial cells. Similar patterns have also been reported when comparing untreated CeD patients with healthy controls using cross-sectional samples (22, 23).

When focusing on patients with or without small intestinal villous atrophy, we found that at follow-up, with the exception of a small set of genes, gene expression signatures were largely comparable between these groups. Notably, although not fulfilling our criteria for diverging gene expression, *IL32* was significantly upregulated (logFC 3.1, FDR 0.01) in patients with atrophy at follow-up, but not in patients without (logFC 1.5, not significant), nor in the general gluten challenge comparison. IL-32 is a pro-inflammatory cytokine, which has also previously been reported to be significantly upregulated after a gluten challenge in CeD (16, 24), but not in cross-sectional studies comparing controls to CeD patients at diagnosis (22, 23). Therefore, it is possible that increased *IL-32* expression may be specific to an acute gluten recall response, such as a challenge, but this requires future verification.

*HLA-DQB1* has previously not been reported to be differentially expressed in bulk RNA sequencing studies of CeD intestine during gluten challenge (16, 24). This discrepancy may be due to differing challenge lengths. *HLA-DQB1* has been reported to be differentially expressed between potential and overt CeD patients (22), and a similar pattern was seen between DH patients with or without villous atrophy at follow-up. This could possibly reflect increased antigen presentation in subjects with villous atrophy compared to subjects with normal mucosal architecture.

Beyond these gene expression changes related to presence or absence of atrophy, we were unable to find genes or clinical factors predicting the villous atrophy on day 0. Stamnaes et al. found that CeD patients undergoing a 14-day gluten challenge presented with distinct proteomic signatures already on day 0, depending on whether the patients were “responders or “non-responders” (25). The “responders” had a lower Vh:CrD than “non-responders” at the end of challenge but also importantly already before the challenge. In our data, the Vh:CrD at day 0 were comparable between those with and without atrophy at follow-up and therefore reasons explaining why only a subset of patients progress to villous atrophy await detailed characterization.

In both dermatological relapse presentations (rash/no rash and dermal IgA/no IgA), changes in gene expression patterns were highly similar, reflecting an expected major overlap between these presentations. Interestingly, *IL10RB* was downregulated and *DOCK8* upregulated in patients with rash or IgA depositions at follow-up, but not in patients without. Neither of these genes have previously been found to be significantly differentially expressed in CeD gluten challenge, and cross-sectional studies have not demonstrated either meaningful log fold change or adjusted *p*-value (22, 23). *IL10RB* mutations are linked to both colitis and early-onset colitis (26, 27), with many of these patients presenting with skin pathology (28). The protein product of *IL10RB* is involved in forming multiple cytokine receptor complexes, which mediate various biological functions including type III IFN signalling, but also immunosuppression when in complex with IL10RA (29). Thus, it can be hypothesized that *IL10RB* downregulation could play a role in dermatological outcomes via immunosuppressive cytokine signalling through receptor complex IL10RB and IL10RA.

*DOCK8,* with increased expression at follow-up in patients with rash or dermal IgA deposition, is predominantly expressed by immune cells and plays a role in immune cell survival, migration, and function (30). Specifically in the intestinal lamina propria, its expression has been shown to be required for IgA-positive plasma cell survival in response to certain antigens (31). Although in CeD small intestinal plasma cells *DOCK8* expression has been reported to be low (32), it is plausible that plasma cells secreting IgA-class TG3 antibodies, found exclusively in DH patients with skin symptoms (4), could contribute to this signal. IELs of CeD patients have also been shown to express *DOCK8* (33), which may be the source of increased expression in our data as well, reflecting the significantly higher amount of CD3^+^ and γδ^+^ IELs in patients with rash. If true, it is, however, unlikely that γδ-IELs directly mediate skin pathology due to lack of clonal relatedness between skin and gut γδ-cells in DH (34).

The strengths of this study include a well-characterized patient cohort, sampling at multiple timepoints, and the possibility to perform temporal analysis on paired samples. Nonetheless, the study has limitations, including the small sample size which prohibited stratification into fully non-overlapping relapse presentation groups, with only atrophy, only rash or only dermal IgA. Additionally, more sample types, such as PBMC samples at follow-up and skin samples could have created more comprehensive understanding of the connection between skin and intestine during challenge.

Furthermore, varying length of the gluten challenge at follow-up may have introduced unwanted heterogeneity into the data, and predetermined biopsy timepoints before relapse would have been ideal. Finally, bulk RNA sequencing presents limitations in terms of determining gene expression profiles of different cell types, which could be mitigated by implementing single cell approaches.

To conclude, we show that although a 3-day gluten challenge does not elicit a marked transcriptomic change in DH PBMCs, a long gluten challenge leads to duodenal gene expression changes very similar to those previously observed in CeD. Despite patients with distinct relapse presentations at follow-up presented with largely parallel transcriptomic responses, we identified gene expression changes of *IL10RB* and *DOCK8,* as well as *HLA-DQB1* and *IL32* specific for dermal relapse and villous atrophy, respectively. The results both confirm pre-conceived notions of the molecular landscape in DH intestine during gluten challenge and provide grounds for further research into the connection between skin and intestine in DH.

## Data Availability

The gene expression and metadata are available on request from the corresponding author within the limits of the ethical approval. The data are not publicly available due to Finnish legislation concerning patient-related data.

## Supplementary material

Appendix S1

Table SI

Figure S1.

Table SII-SXVIII
