# Supplementary material for: Connecting Skin and Gut in Dermatitis Herpetiformis: Insights from Gluten Challenge Transcriptomics
Source: Acta Derm Venereol. 2026 Sep 9;106:0792. doi: 10.2340/actadv.v106.adv-2026-0792 (PMC13558545; doi:10.2340/actadv.v106.adv-2026-0792)
Supplement: Supplementary file 1 [file ActaDv-106-0792-s0001.pdf]

Supplementary Materials and methods

RNA extraction from intestinal biopsies

The biopsies were first washed with 200ul ice cold PBS, followed by 1-minute vortex, 10 min incubation on ice, and 2-minute vortex. The resulting supernatant was saved for microbial DNA extraction. Thereafter the biopsies were transferred into bead-beating tubes together with 600ul RLT buffer (Qiagen, Hilden, Germany), followed by 1 x 30sec 6m/s homogenization in a FastPrep Tissue homogenizer (MP Biomedicals, CA, United States). The tubes were centrifuged 5 min (1000 g, 4 °C) and the supernatant was transferred into an eppendorf tube. The bead-beating homogenization was repeated by adding 400 ul RLT buffer to the tube followed by 2 x 30sec 6m/s homogenization in FastPrep Tissue homogenizer, and centrifugation as above. The resulting supernatant was combined with the supernatant from the first round. RNA purification from the supernatant was carried out using the RNeasy Mini kit (Qiagen, Hilden, Germany) according to manufacturer's instructions.

Bulk RNA sequencing library construction of PBMC and intestinal biopsy samples

Sequences for primers and indexes used in sequencing are all listed in Table S2 (Supplemenatry Tables). Briefly, 10 or 20 ng of RNA was mixed with reverse transcriptase mix containing 10 mM dNTPs (Meridian Bioscience, Cincinnati, OH, United States), RNase inhibitor (Thermo Fisher Scientific, Waltham, MA, United States), 50 µM template switch oligo (Integrated DNA Technologies, Coralville, IO, United States), reverse transcriptase (Thermo Fisher Scientific, Waltham, MA, United States), 5 M betaine (Sigma-Aldrich, St.Louis, MO, United States), and RNase free water (Qiagen, Hilden, Germany). Poly-A capturing indexing oligos (Integrated DNA Technologies, Coralville, IO, United States) at 2 µM concentration were added to the mix and reverse transcriptase reaction was carried out in a T100 thermocycler (Bio-Rad, Hercules, CA, United States) at 21°C for 30 min followed by 52°C for 90 min. Following the reverse transcriptase reaction, unwanted products such as primers were digested with Exonuclease 1 (Thermo Fisher Scientific, Waltham, MA, United States) for 45 min in 37 °C followed by inactivation of the enzyme at 85 °C for 15 min. The resulting cDNA was amplified in a PCR reaction using KAPA HiFi HotStart ReadyMix (Roche, Basel, Switzerland) and 200uM SMART PCR primer (Integrated DNA Technologies, Coralville, IO, United States) in a T100 thermocycler with the following cycles: 95°C for 3 min, 4 cycles of 98°C for 20 s, 65°C for 45 s, 72°C for 3min, then 12 cycles of 98°C for 20s, 67°C for 20s, and 72°C for 3 min. Final extension step was carried out in 72°C for 5 min. The PCR products were pooled and purified using 0.6x HighPrep PCR Clean up system (MagBio Genomics, Gaithersburg, MD, United States) according to manufacturer's instructions and eluted to 30 µl nuclease-free water. The libraries were tagged for 3' sequencing with the Nextera XT DNA Library Prep Kit tagmentation reaction (Illumina, San Diego, CA, United States). 1 ng of cDNA served as input for the tagmentation reaction, to which also a P5 primer and Nextera N7xx indexes were added (Integrated DNA Technologies, Coralville, IO, United States). The tagmented products were purified twice using 0.6x HighPrep PCR Clean up system and eluded to 25 µl nuclease-free water. The quality of the libraries was checked using the LabChip GXII Touch HT electrophoresis system (PerkinElmer, Waltham, MA, United States) or Tapestation (Agilent, SC, California, United States), the concentration of the libraries was measured with Qubit 2 fluorometer (Invitrogen, Waltham, MA, United States). The initial libraries produced from intestinal samples were found to be uneven in composition between day 0 and end of follow-up samples, likely due to varying RNA quality. Therefore, the libraries were to be constructed and sequenced a second time. The reads from each sequencing run were combined using the sumTechReps function in edgeR[1].

Sequencing

Libraries were sequenced using the Nextseq 500 instrument (Illumina, San Diego, CA, United States), using a custom read 1 primer. The produced libraries consisted of 20 bp read 1 and 55bp (pair-end) read 2. NextSeq high Output 1x75bp kits (Illumina, San Diego, CA, United States) were used for both PBMC and intestinal libraries, resulting in sequencing depth of 16.5 million reads/sample for PBMC samples, and 12 million

reads/sample for intestinal samples. Sequencing was performed at the Functional Genomics Unit of the University of Helsinki, Finland.

Analysis of bulk RNA sequencing data
FASTQ-files from the bulk RNA sequencing libraries were processed into a digital gene expression matrix following the published guidelines, also described by Freitag et al. (2020) [2]. Read 1 and Read 2 were first merged into one FASTQ file, with trimming of adapter and poly-A sequences. The merged reads were aligned to the primary assembly GRCh38 human genome (patch release 14) using STAR aligner [3]. Aligned reads were further demultiplexed and the digital expression matrix was created. Altogether, four samples were removed from the differential gene expression analysis due to low quality. RStudio version 4.4.1[4] was used for statistical and downstream analyses of the data. In RStudio, edgeR and limma [5] were used for the differential gene expression analysis, whereas fgsea [6] using datasets from The Molecular Signatures Database [7,8] was applied for gene set enrichment analysis. FDR was controlled using the Benjamini–Hochberg method. Specifically, pathway collections Gene Ontology biological process [8,9], Reactome [10], and KEGG Legacy [11] gene sets were used. In addition, from the cell type signature sets, gene signatures defining small intestinal cells [12] was used. Genes ranks were assigned as signed p-values. Packages ComplexHeatmap [13] and tidyverse [14] were used in result visualization.

For the PBMC data, hemoglobin genes were removed using the grepl pattern "^HB[^(P)]" before further downstream analyses.

Supplementary References

1 Robinson MD, McCarthy DJ, Smyth GK. edgeR: a Bioconductor package for differential expression analysis of digital gene expression data. Bioinformatics 2010; 26:139–40.

2 Freitag TL, Podojil JR, Pearson RM, Fokta FJ, Sahl C, Messing M, et al. Gliadin Nanoparticles Induce Immune Tolerance to Gliadin in Mouse Models of Celiac Disease. Gastroenterology. 2020 May 1;158(6):1667-1681.e12. doi:10.1053/j.gastro.2020.01.045

3 Dobin A, Davis CA, Schlesinger F, Drenkow J, Zaleski C, Jha S, et al. STAR: ultrafast universal RNA-seq aligner. Bioinformatics. 2013 Jan 1;29(1):15–21. doi:10.1093/bioinformatics/bts635

4 Posit team (2025). RStudio: Integrated Development Environment for R. Posit Software, PBC, Boston, MA. URL http://www.posit.co/.

5 Ritchie ME, Phipson B, Wu D, et al. limma powers differential expression analyses for RNA-sequencing and microarray studies. Nucleic Acids Res 2015; 43:e47.

6 Korotkevich G, Sukhov V, Budin N, et al. Fast gene set enrichment analysis. 2021; :060012.

5 Liberzon A, Subramanian A, Pinchback R, et al. Molecular signatures database (MSigDB) 3.0. Bioinformatics 2011; 27:1739–40.

7 Subramanian A, Tamayo P, Mootha VK, et al. Gene set enrichment analysis: A knowledge-based approach for interpreting genome-wide expression profiles. Proc Natl Acad Sci 2005; 102:15545–50.

8 Ashburner M, Ball CA, Blake JA, et al. Gene Ontology: tool for the unification of biology. Nat Genet 2000; 25:25–9.

9 The Gene Ontology Consortium, Aleksander SA, Balhoff J, et al. The Gene Ontology knowledgebase in 2023. Genetics 2023; 224:iyad031.

10 Jassal B, Matthews L, Viteri G, et al. The reactome pathway knowledgebase. Nucleic Acids Res 2020; 48:D498–503.

11 Kanehisa M, Sato Y, Kawashima M, et al. KEGG as a reference resource for gene and protein annotation. Nucleic Acids Res 2016; 44:D457–62.

12 Busslinger GA, Weusten BLA, Bogte A, et al. Human gastrointestinal epithelia of the esophagus, stomach, and duodenum resolved at single-cell resolution. Cell Rep 2021; 34:108819.

13 Gu Z. Complex heatmap visualization. iMeta 2022; 1:e43.

14 Wickham H, Averick M, Bryan J, et al. Welcome to the Tidyverse. J Open Source Softw
