# Supplementary material for: Connecting Skin and Gut in Dermatitis Herpetiformis: Insights from Gluten Challenge Transcriptomics
Source: Acta Derm Venereol. 2026 Sep 9;106:0792. doi: 10.2340/actadv.v106.adv-2026-0792 (PMC13558545; doi:10.2340/actadv.v106.adv-2026-0792)
Supplement: Supplementary file 2 [file ActaDv-106-0792-s0002.pdf]

Table SI. Key characteristics of samples involved in the study

	**PBMC samples all**	**Intestinal samples all**	**Intestinal samples paired**
**Day 0 sample, n**	12	16	11
Villous atrophy at FE, n	10	13	8
Rash at FE, n	10	12	7
IgA deposits at FE, n	8	10	6
**Day 6 sample n**	11	*NA*	*NA*
Villous atrophy at FE, n	9	-	-
Rash at FE, n	8	-	-
IgA deposits at FE, n	7	-	-
**FE sample n**	*NA*	13	11
Villous atrophy at FE, n	-	9	8
Rash at FE, n	-	9	7
IgA deposits at FE, n	-	7	6

Number of patient samples with given clinical symptoms at FE denoted for each type of sample for each sampling timepoint. Intestinal samples are further divided into analysis of all samples and analysis of paired samples only. Abbreviations: PBMC, peripheral blood mononuclear cells; NA, not available; FE, end of follow-up.
