# Supplementary material for: Connecting Skin and Gut in Dermatitis Herpetiformis: Insights from Gluten Challenge Transcriptomics
Source: Acta Derm Venereol. 2026 Sep 9;106:0792. doi: 10.2340/actadv.v106.adv-2026-0792 (PMC13558545; doi:10.2340/actadv.v106.adv-2026-0792)

Fig. S1 Principal component analysis of dermatitis herpetiformis patient small intestinal biopsy RNA expression in response to gluten challenge. PC, principal component.

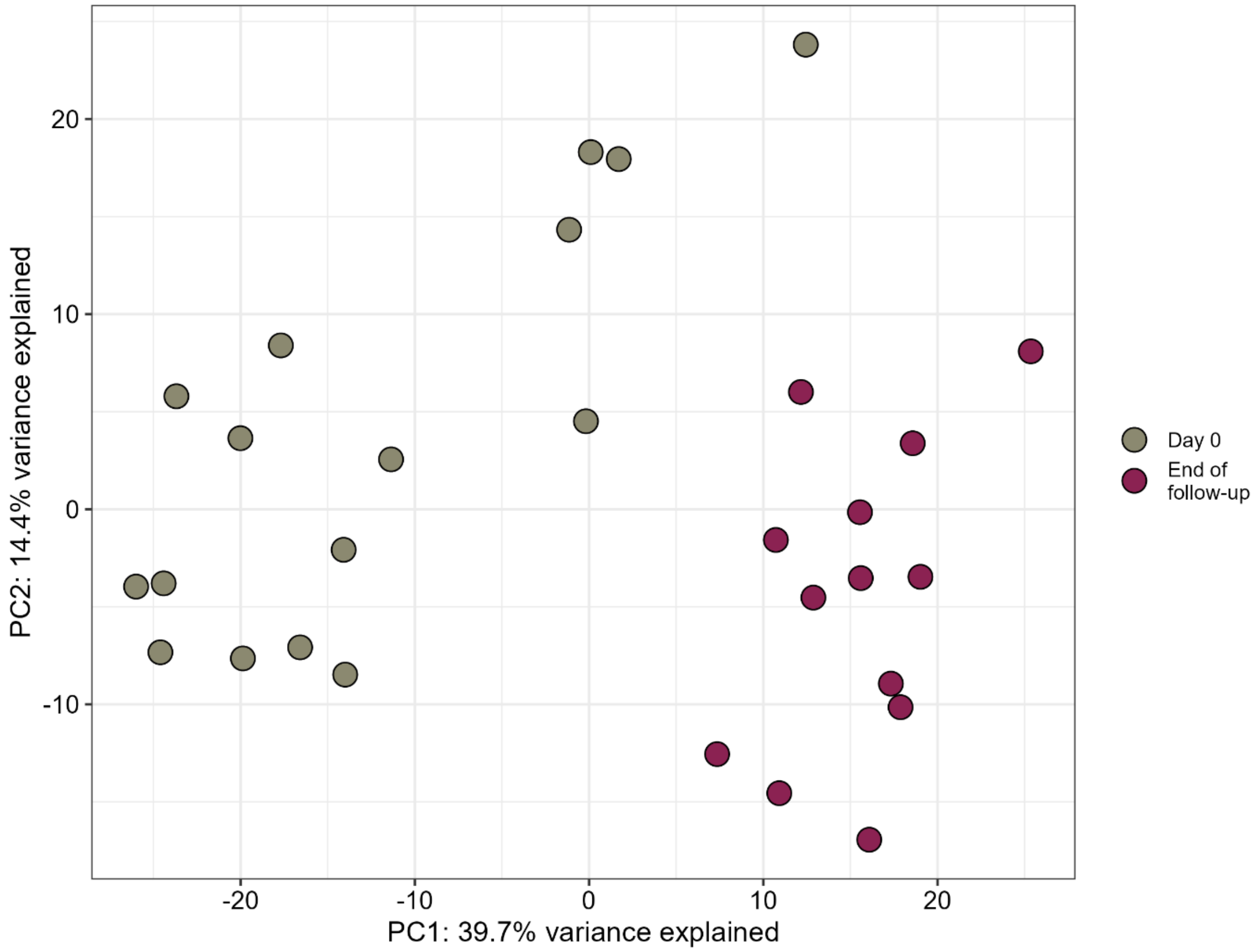

Supplement: Supplementary file 3 [file ActaDv-106-0792-s0003.pdf]
